# Effect of Resident Physicians in a Supervisory Role on Efficiency in the Emergency Department

**DOI:** 10.5811/westjem.2020.7.46587

**Published:** 2020-08-24

**Authors:** Aaron S. Kraut, Lauren Sheehy, Benjamin H. Schnapp, Brian Patterson

**Affiliations:** * University of Wisconsin School of Medicine and Public Health-BerbeeWalsh, Department of Emergency Medicine, Madison, Wisconsin; † University of Wisconsin-Madison, Department of Industrial and Systems Engineering and Department of Biostatistics and Medical Informatics, Madison, Wisconsin

## Abstract

**Introduction:**

While patient throughput and emergency department (ED) length of stay (LOS) are recognized as important metrics in the delivery of efficient care, they must be balanced with the educational mission of academic centers. Prior studies examining the impact of learners on throughput and LOS when staffing directly with attending physicians have yielded mixed results. Herein we sought to examine the impact of a staffing model involving a supervisory resident “pre-attending” (PAT) on ED throughput and LOS, as this model offers a valuable educational experience for residents, but may do so at the expense of operational efficiency.

**Methods:**

We retrospectively analyzed 26,702 unique patient encounters at a university-affiliated community ED between July 1, 2017–January 1,2019. The experimental group was comprised of patients seen primarily by midlevel providers, who staffed with a PAT, who subsequently staffed with an attending physician. The control group was comprised of patients seen by midlevel providers and staffed directly with attendings without a PAT. We used a parametric hazard model to analyze the effect of the presence of a PAT on service time, controlling for potential confounders including timing of presentation and patient demographics.

**Results:**

The presence of a PAT is associated with a statistically significant increase in service time of five minutes (p = 0.006). Holding other variables equal, predicted service time in the experimental group was 173 minutes (95% confidence interval (CI), 171–176), while that for controls was 168 minutes (95% CI, 165–171).

**Conclusion:**

The presence of a PAT is associated with a statistically significant increase in service time, but the magnitude (five minutes) is likely operationally insignificant. The negligible increase in service time is offset by the benefit to residents’ training. The results of this study may be helpful for residency programs considering the addition of a PAT shift structure.

## INTRODUCTION

Patient throughput and emergency department (ED) length of stay (LOS) are recognized as important metrics in the delivery of efficient care in emergency medicine (EM).^1^ However, academic centers must balance expeditious care delivery with the educational mission of training the next generation of emergency physicians.^2^ Often, educational leaders must overcome operational resistance to learning initiatives that may threaten clinical efficiency without sufficient data to justify the implementation of their educational strategies. This is particularly germane to discussions about teaching and supervisory structures in the ED.

Prior studies have reported conflicting evidence on the effect of learners on ED patient throughput. Bhat et al demonstrated that attending physicians’ patients per hour were increased when working with a resident learner compared to working alone, suggesting increased efficiency.^3^ Conversely, several recent studies have reported positive correlations between the presence of residents and ED LOS.^4^,^5^,^6^ However, multiple additional studies have demonstrated that ED LOS is unaffected by the presence of residents or medical students.^7^,^8^

The addition of a supervisory “pre-attending” role (PAT) provides a unique and valuable educational experience for residents late in their training and addresses the Accreditation Council for Graduate Medical Education (ACGME) directive to incorporate graduated responsibility into residency training.^9^ Under a standard patient care model, a patient is evaluated by a resident or an advanced practice provider (APP), who is supervised directly by an attending physician. With the supervisory PAT model, a patient is evaluated by an APP, who is supervised by a PAT resident, who is then supervised by an attending physician.^10^ This provides senior residents the opportunity to supervise care in a controlled setting that mimics the environment in which they will practice upon graduation from residency. In addition, this care delivery model is recommended by the Society of Emergency Medicine Physician Assistants and endorsed by the American College of Emergency Physicians as a means of preparing residents to be leaders of physician-APP teams in clinical practice.^11^

Our previous work in developing the PAT experience using mastery learning principles suggests that resident participants feel it is highly educationally valuable.^12^ However, the effect of this care delivery model on ED patient throughput has not yet been examined. Given the staffing model’s educational value, understanding how the presence of a PAT affects patient throughput is critical for educators in EM seeking to justify this implementation of a graduated responsibility model for their trainees.

Including an additional provider in a patient’s evaluation has the potential to increase ED LOS by adding another individual who must interview and examine the patient, but it could also expedite patient workups if the PAT is able to provide attending-level oversight to APPs, essentially doubling the “attending” coverage in the ED. We sought to determine the effect of a supervisory resident PAT on the clinical efficiency of a university-affiliated community ED. In addition, we endeavored to quantify and qualify the educational value of the PAT experience for resident physicians.

## METHODS

### Study Design

We conducted a retrospective observational study using a dataset of consecutive patients from a single, university-affiliated, community ED with approximately 18,000 visits per year. Relevant variables were extracted from the electronic health record (EHR) (Epic, Verona, WI) via data query. All patients who presented from July 1, 2017–January 1, 2019, during the days of the week and hours when the PAT may have been working were included in the analysis. This study was reviewed by the institutional review board and declared exempt.

### Study Setting and Population

The study ED is covered by attending physicians in 12-hour, single covered shifts from 7 am–7 pm. APPs covered three shifts from 9 am–5 pm, 12 pm–9 pm, and 5 pm–2 am. Additionally, during weeks when a PAT resident was scheduled, that resident would work from 9 am–7 pm Monday, Tuesday, Wednesday and Friday. In this way, we abstracted data on patients who presented from 9–5 on these days in our analysis. The experimental group was comprised of patients seen by APPs and attendings with a supervisory PAT resident. These PAT supervisory residents were third-year EM residents in a three-year academic EM residency program. The control group consisted of those patients seen by APPs and attendings without a PAT supervisory resident.

### Measurements

Analysis was conducted on data abstracted from the EHR. We determined PAT status by presence or absence of a PAT assigned to the patient’s treatment team. Self-assignment to the treatment team is a standard part of the PAT workflow for all residents. The EHR records the time of patient rooming as well as the time when the patient is dispositioned (as determined by an order to admit, transfer, or discharge the patient). LOS, our primary outcome, was calculated as the difference between these two times. The following variables were abstracted for each patient encounter: age; gender; hour of day; day of week; and disposition. These variables were preselected for analysis in advance based on both likelihood of potentially affecting patient LOS and potential to vary between PAT and non-PAT shifts.

### Data Analysis

We used a parametric hazard model to examine the association between the explanatory variables and the pickup time. Analyses were conducted using Stata 14 (Stata Corp, College Station, TX). We analyzed LOS as a time-to-event outcome using a parametric proportional hazard model.^13^ This model assumes an underlying functional form of the duration distribution and then estimates the multiplicative or proportional effect of each explanatory variable on the underlying distribution.^14^ We tested six underlying distributions (exponential, Weibull, gompertz, lognormal, log logistic, and generalized gamma), and while the results were qualitatively similar between the models we found the gamma distribution provided the best model fit based on both Akaike and Bayesian information criteria.^15^ All model results presented are based on the gamma distribution model.

## RESULTS

We analyzed a total of 26,702 patient encounters that occurred within the specified date range. Of these encounters, two were dropped for missing data (no roomed time), 328 were dropped based on nonstandard disposition (ie, discharge against medical advice; left without being seen), and a further 18,424 were removed due to taking place on nights or weekends when a PAT is never scheduled. The remaining 7948 encounters were divided into PAT and non-PAT. Control variables are displayed by PAT status in the Table.

For the 4527 PAT encounters, the unadjusted mean LOS was 190 minutes (95% confidence interval [CI], 187-193). For the 3421 non-PAT encounters, the unadjusted LOS was 180 minutes (95% CI, 177–183). In the parametric hazard model, presence of a PAT was significantly associated with a marginal LOS increase of five minutes: the adjusted mean LOS for PAT encounters was 173 minutes (95% CI, 171–176) and the adjusted mean LOS for non-PAT encounters was 168 minutes (95% CI, 165–171; Table).

## DISCUSSION

Our study demonstrated that resident PATs supervising APPs do have a small but significant effect on time to disposition for patients in the ED. With all EM residency programs balancing the dual mandate of resident education and care for patients, this data can be helpful as programs consider whether and how to implement supervising resident roles.

While it may seem intuitively obvious that requiring an additional physician to evaluate each ED patient adds to the LOS, this is not necessarily the case. Previous studies have shown that consultant evaluation, imaging and laboratory tests are some of the most significant factors impacting time in the ED.^16^ If the PAT had been seeing patients while he or she was waiting for these tests, it is possible that we would have seen no effect on ED LOS. The effect of supervising residents would also likely be washed out in clinical environments with long wait times. As this study was conducted in a community ED with virtually no wait times and a relatively high percentage of simple complaints like ear infections, this likely was a contributing factor to our findings. However, while the increased LOS we found was statistically significant, it is not clear whether this was operationally important, as it represents only a 3% increase over the average LOS for encounters that do not involve the PAT. Further, previous operations literature on ED LOS has described an improvement of 11 minutes as “modest,” suggesting that effects on ED crowding are likely to be minimal.^17^

The ACGME requires all residencies to implement graduated responsibility; trainees cannot simply see a greater number of patients as they progress in training but must be entrusted with more roles and tasks as they progress.^9^ While a PAT role is only one way of addressing this mandate, this role has high appeal to both residents and teaching faculty because it mimics as closely as possible the experience nearly all graduates will have when they leave residency and first become an attending: supervising APPs, residents, or medical students. In our previously published work in developing a mastery learning curriculum for the PAT role, we reported that 75% of participating residents felt more prepared to function as an attending because of their PAT experience, while 66% agreed that they learned things in the PAT role that they would not have otherwise. The majority of participants also reported that the feedback they received in the PAT role helped them to improve as physicians and aided in their ability to secure a job after completion of residency^12^ (Appendix A). Anecdotally, many residents identified the PAT experience as “highly valuable” in comments from their semi-annual evaluations and consistently highlight the role as one of our program’s greatest strengths on our Annual Program Evaluation survey. “A great improvement to third year,” wrote one graduate. “It was an eye opening experience staffing the APPs, and I was asked about it at every single one of my job interviews.”

In addition to added educational value, the PAT role can assist educational leaders in the realm of resident assessment. The Emergency Medicine Milestones set out a variety of competencies in which programs are required to assess residents’ progress; a PAT role can allow the assessment of skills such as task switching and multitasking in a closely supervised setting.^18^

## LIMITATIONS

This was a retrospective, single-center study that occurred at a community ED (although affiliated with an academic site), where APPs and students were supervised by the PAT. Conclusions may not be generalizable to an academic setting where the supervising resident would supervise junior residents. It is possible that other variables, such as increasing patient volumes over time, may have contributed to our findings. This study focused on LOS as a measure of quality of care and did not assess other patient care outcomes that may be affected by differing staffing structures, such as relative value units, number of tests ordered, or number of return visits. Importantly, this study did not rigorously assess objective learning outcomes associated with the PAT model of care delivery, such as the achievement of ACGME Milestone benchmarks, nor did it systematically evaluate APP satisfaction with this model. These both represent ideal outcome measures for future studies.

## CONCLUSION

The presence of a “pre-attending” is associated with an increase in time to disposition of 5 minutes. The downsides of this 3% increase in time to disposition are likely outweighed by the significant benefits to residents’ training, which, although subjectively significant, could be assessed by more objective measures in future studies. The results of this study may serve as critical justification for residency programs seeking to implement a graduated supervisory structure in the face of concerns about adverse effects on patient throughput and operational efficiency.

## Supplementary Information



## Figures and Tables

**Table:** Patient demographics and mean length of stay for control (non-pre-attending encounters) and experimental (pre-attending encounters) groups.

	Pre-attending encounters N = 4,527 (95% CI)	Non-pre-attending encounters N = 3,421 (95% CI)
Age	47.7(47.1–48.4)	45.8(45.0–46.5)
Female gender	57.8(56.3–59.2)	54.4(52.7–56.0)
Proportion discharged	78.4(77.2–79.5)	80.5(79.1–81.8)
Unadjusted LOS (min)	190 (187–193)	180(177–183)
Adjusted LOS (min)	173 (171–176)	168(165–171)

*CI,* confidence interval*; PAT,* pre-attending; *LOS,* length of stay; *min,* minutes.
