# Supplementary material for: Effect of Resident Physicians in a Supervisory Role on Efficiency in the Emergency Department
Source: West J Emerg Med. 2020 Aug 24;21(5):1266–9. doi: 10.5811/westjem.2020.7.46587 (PMC7514401; doi:10.5811/westjem.2020.7.46587)
Supplement: Supplementary file 1 [file wjem-21-1266-s001.docx]

**Appendix A**

Survey of PGY3 EM residents on the value of the PAT role.

1. The PAT experience so far was a good use of my time.

Strongly Agree

Agree

Neutral

Disagree

Strongly Disagree

2. I have learned a lot in the PAT role that I would not have learned otherwise.

Strongly Agree

Agree

Neutral

Disagree

Strongly Disagree

3. I feel more prepared to be an attending next year because of the PAT role.

Strongly Agree

Agree

Neutral

Disagree

Strongly Disagree

4. The feedback I received in the PAT role helped me improve as a physician.

Strongly Agree

Agree

Neutral

Disagree

Strongly Disagree

5. My experience as a PAT helped me get a job for next year.

Strongly Agree

Agree

Neutral

Disagree

Strongly Disagree

6. Please provide any feedback for improvement of the PAT role going forward. (free response)
